# The Arabidopsis EAR-motif-containing protein RAP2.1 functions as an active transcriptional repressor to keep stress responses under tight control

**DOI:** 10.1186/1471-2229-10-47

**Published:** 2010-03-16

**Authors:** Chun-Juan Dong, Jin-Yuan Liu

**Affiliations:** 1 Laboratory of Molecular Biology and MOE Laboratory of Protein Science, School of Life Sciences, Tsinghua University, Beijing 100084, China

## Abstract

**Background:**

Plants respond to abiotic stress through complex regulation of transcription, including both transcriptional activation and repression. Dehydration-responsive-element binding protein (DREB)-type transcription factors are well known to play important roles in adaptation to abiotic stress. The mechanisms by which DREB-type transcription factors activate stress-induced gene expression have been relatively well studied. However, little is known about how DREB-type transcriptional repressors modulate plant stress responses. In this study, we report the functional analysis of RAP2.1, a DREB-type transcriptional repressor.

**Results:**

RAP2.1 possesses an APETALA2 (AP2) domain that binds to dehydration-responsive elements (DREs) and an ERF-associated amphiphilic repression (EAR) motif, as the repression domain located at the C-terminus of the protein. Expression of *RAP2.1 *is strongly induced by drought and cold stress via an ABA-independent pathway. Arabidopsis plants overexpressing *RAP2.1 *show enhanced sensitivity to cold and drought stresses, while *rap2.1-1 *and *rap2.1-2 *T-DNA insertion alleles result in reduced sensitivity to these stresses. The reduced stress sensitivity of the plant containing the *rap2.1 *allele can be genetically complemented by the expression of *RAP2.1*, but not by the expression of EAR-motif-mutated *RAP2.1*. Furthermore, chromatin immunoprecipitation (ChIP) analysis has identified *Responsive to desiccation/Cold-regulated *(*RD/COR*) genes as downstream targets of RAP2.1 *in vivo*. Stress-induced expression of the *RD/COR *genes is repressed by overexpression of *RAP2.1 *and is increased in plants expressing the *rap2.1 *allele. In addition, RAP2.1 can negatively regulate its own expression by binding to DREs present in its own promoter. Our data suggest that RAP2.1 acts as a negative transcriptional regulator in defence responses to cold and drought stress in Arabidopsis.

**Conclusions:**

A hypothetical model for the role of RAP2.1 in modulating plant responses to cold and drought is proposed in this study. It appears that RAP2.1 acts as a negative "subregulon" of DREB-type activators and is involved in the precise regulation of expression of stress-related genes, acting to keep stress responses under tight control.

## Background

Drought, cold and high salinity are the major adverse environmental factors that can adversely affect plant growth and crop production. A variety of genes are induced under these stress conditions, enabling plants to adapt to these abiotic stresses [1]. It is well known that complex transcriptional regulatory networks are involved in stress-induced changes in gene expression [1]. Among the best characterized stress-responsive transcription factors are the dehydration responsive element (DRE) binding proteins DREBs [2-4]. The DREB protein family can be divided into six small groups (A-1~A-6) based on similarity in the APETALA2 (AP2) DNA-binding domain [5]. Most reports have focused on DREB-type transcriptional activators. Three DREB1 proteins, DREB1A, DREB1B, and DREB1C, members of the A-1 DREB group, transactivate cold-induced expression of *RD/COR/LTI *(*responsive to dehydration/cold-responsive/low-temperature-induced*) genes through interactions between their AP2 DNA binding domains and the core DRE *cis*-elements (A/GCCGAC) present in the promoters of the target genes [2,4,6]. Overexpression of each *DREB1 *constitutively induces the DREB1 regulon and enhances plant freezing tolerance [7,8]. Similar results have been reported for the constitutive active form of the DREB2 proteins, the A-2 group members, under dehydration and high salinity stress conditions [3,9]. TINY, a member of the A-4 DREB group, can activate the expression of both DRE- and ERE- (for ethylene responsive element) regulated genes. In this way, TINY plays a role in the crosstalk between abiotic and biotic stress-responsive gene expression pathways by connecting the DRE- and ERE-mediated signaling pathways [10]. RAP2.4, a member of the A-6 group, functions as a transactivator of DRE- and ERE-mediated genes that are responsive to light, ethylene and drought, suggesting that RAP2.4 acts in the cross-talk between the light and ethylene signaling pathways to coordinately regulate multiple development processes and stress responses [11].

Although the mechanisms of activation mediated by DREB proteins involved in plant stress responses are relatively well studied, little is known about the negative regulation of stress genes mediated by the DREB-type transcriptional repressors. Transcriptional repression is an essential mechanism in the precise control of gene expression [12]. Transcriptional repressors may maintain the stress response genes in an off state in the absence of any stress. In addition, they may keep the expression of stress response genes under tight control, to prevent the metabolic waste and self-inflicted damage that can be caused by a runaway stress response [13].

In plants, transcriptional repressors containing the ERF-associated amphiphilic repression (EAR) motif have been reported to play important roles in modulating plant stress and defense responses [13]. The EAR-motif [^L^/_F_DLN^L^/_F_(x)P] was first identified in the C-terminal region of class II ERFs (Ethylene Response Factor) and C2H2- (Cys2/His2) type zinc-finger proteins [14]. Recently, many studies have revealed the *in planta *roles of EAR-motif-containing repressors in modulating plant responses to drought [15-17], cold [16-18], UV [19], pathogen infection [20], and hormone signaling [15,21,22]. The EAR-repressor AtERF4 binds to the GCC box of *PDF1.2*, a gene encoding an antimicrobial peptide, and represses its jasmonate-ethylene-dependent expression. Overexpression of *AtERF4 *in Arabidopsis renders the plants more susceptible to the wilt pathogen *Fusarium oxysporum *[20]. Similar to AtERF4, AtERF7 binds to the GCC box of ABA-induced genes and represses their transcription. Arabidopsis plants overexpressing *AtERF7 *show a reduced sensitivity of guard cells to ABA and an increase in transpired water loss [15]. Another example of EAR-repressors are the key members of the C2H2 zinc-finger family of proteins, such as ZAT7 [23], ZAT10 [16] and ZAT12 [17,18]. These proteins suppress the repressors of defense responses, thus increasing Arabidopsis tolerance to abiotic stress.

The first DREB-type transcriptional repressor identified was found in *Gossypium hirsutum*, as GhDBP1, a member of the A-5 DREB group [23]. GhDBP1 can specially bind to the DRE and repress the expression of a reporter gene driven by DRE in tobacco leaves. The transcriptional repression domain utilized by GhDBP1 is located in the EAR-motif-like domain in the C-terminal region of the protein [24]. This domain is also found in other DREB proteins, including RAP2.1 from Arabidopsis, GmDREB1 from soybean, and OsRAP2.1 from rice [23]. These findings suggest that there may be a molecular adaptation mechanism in plant stress responses, harmoniously mediated by DREB proteins that function as either activators or repressors. This expectation provokes our interest in exploring the corresponding molecular behaviors of DREB-type transcription repressors in plants.

This study establishes that RAP2.1 is a DREB-type, EAR-motif-containing transcriptional repressor that negatively regulates plant responses to cold and drought stresses. This repression by RAP2.1 maintains tight control over these responses. In Arabidopsis, RAP2.1 is transcriptionally activated by drought and cold stresses and binds to the DRE/CRTs in the promoters of *RD/COR *genes, repressing the stress-induced expression of such genes. Arabidopsis plants overexpressing *RAP2.1 *show enhanced sensitivity to cold and drought stresses, whereas *rap2.1 *T-DNA insertion alleles result in reduced stress sensitivity. Also, we present evidence that RAP2.1 can bind to the DREs present in its own promoter and repress its own expression, indicating a negative feedback control in the regulation of *RAP2.1*'s expression. Together, our findings indicate how RAP2.1, by cooperating with other DREB-type transcriptional activators, modulates plant responses to cold and drought stresses.

## Results

### Sequence characterization of the RAP2.1 gene

**Figure 1:**
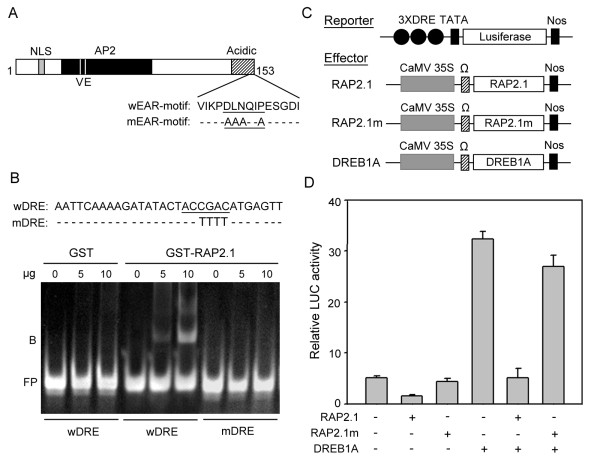
**RAP2.1 binds to DRE and acts as a transcriptional repressor**. (A) Schematic representation of the RAP2.1 amino acid sequence. A nuclear localization signal (NLS), AP2 DNA-binding domain (AP2), a putative acidic domain (Acidic) and the conserved valine (V) and glutamic acid (E) residues are indicated. The key residues of the EAR-motif without (wEAR-motif) or with site-mutation (mEAR-motif) are also shown. (B) RAP2.1 binding to the DRE element. The oligo-nucleotide probes of wild type DRE (wDRE) and mutated DRE (mDRE) used in gel shift assay are listed. DNA probe alone (100 ng) or incubated with 5 μg or 10 μg of recombinant protein were assayed. FP: free probes; B: DNA-protein complex. (C) Diagram of reporter and effector constructs. Ω, translational enhancer of tobacco mosaic virus; Nos, terminator signal of the gene for nopaline synthase. (D) Repression of reporter gene activity by RAP2.1 and suppression of DREB1A-mediated transactivation by RAP2.1. Values shown are means of data taken from three independent experiments; error bars indicate SD.

The DNA sequence of *RAP2.1 *gene was first identified by Okamuro et al. [25]. The 836 bp of the full-length cDNA contains an open reading frame encoding a protein of 153 amino acids, with a predicted molecular mass of 17.2 kDa and a calculated p*I *of 9.82. Examination of the RAP2.1 protein sequence, using programs PROSITE [26] and PredictNLS [27], identified a basic amino acid stretch (^10^MRKRRQ^15^) in its N-terminus that resembles a classical nuclear localization signal (NLS) [27]. RAP2.1 nuclear import could be mediated by its NLS, as is the case for many transcription factors, such as RAP2.4 from Arabidopsis [11], OsWRKY31 from rice [28], and GhDBP1 from cotton [29]. In addition to the NLS sequence, RAP2.1 also contains a typical AP2 DNA-binding domain and an acidic region in its C-terminus, which might act as a transcriptional regulatory domain (Figure 1A, see Additional file 1: Figure S1). The AP2 domain contains conserved valine (V) in the 14th position and glutamic acid (E) in the 19th position, both of which have been reported as conserved in the DREB subfamily [29]. Alignment of RAP2.1 against various AP2/ERF proteins revealed that RAP2.1 also contains another conserved domain, DLNxxP (Figure 1A, see Additional file 1: Figure S1). This domain is very similar to the EAR motif [^L^/_F_DLN^L^/_F_(x)P], which has been identified in many transcriptional repressors of various species [13], suggesting that the RAP2.1 might function as a DREB-type transcriptional repressor in Arabidopsis.

### RAP2.1 binds to the DRE element and acts as a transcriptional repressor

To examine whether RAP2.1 could interact specifically with the DRE motif, we expressed the N-terminal 120 aa of the RAP2.1 protein (containing the AP2 DNA-binding domain) as a GST fusion in *E. coli*, and the purified recombinant proteins were then used for gel mobility shift assays. As shown in Figure 1B, the wild-type DRE (wDRE) interacted with the GST-RAP2.1 fusion protein and was retarded on the gel (lanes 4-6). In contrast, no retardation band was detected for the oligonucleotide harboring the mutant version of the DRE element (mDRE, lanes 7-9). As a control, GST was shown not to bind with wDRE (lanes 1-3). These results suggested that the RAP2.1 protein could bind specifically to the DRE element *in vitro*. However, it is more important to determine whether DRE-binding activity correlates with the transcriptional activity of RAP2.1 *in vivo*.

As mentioned above, RAP2.1 contains a conserved sequence (KPDLNQIP) similar to the EAR-motif, which has been reported as a transcriptional repression domain [13,29]. To determine whether RAP2.1 was capable of repressing DRE-mediated transcription, we performed transient expression assay in Arabidopsis leaves using a reporter gene containing three copies of the DRE sequence from the *RD29A *promoter, 3×DRE-FLUC (Figure 1C). As shown in Figure 1D, expression of *RAP2.1 *resulted in a substantial reduction of the expression of the reporter gene FLUC. Further, DREB1A, a well-known Arabidopsis transcriptional activator [15], induced activation of FLUC by about 7-fold, but co-expression of *RAP2.1 *prevented this activation (Figure 1D). To determine whether the conserved EAR-like-motif was important for the RAP2.1-mediated repression, site-specific mutations were made to convert four conserved amino acids (D_143_L_144_N_145_QIP_148_) to alanines (AAAQIA) (Figure 1A). As expected, the ability of RAP2.1 to repress transcription was abolished when the EAR-motif was mutated (Figure 1D). Together, these results suggest that RAP2.1 may function as a transcriptional repressor, and an intrinsic repression domain exists in the C-terminal EAR-motif, which contains four conserved amino acids (D, L, N, and P) important for the repression activity of RAP2.1.

### RAP2.1 expression is greatly induced by cold and drought stresses

**Figure 2:**
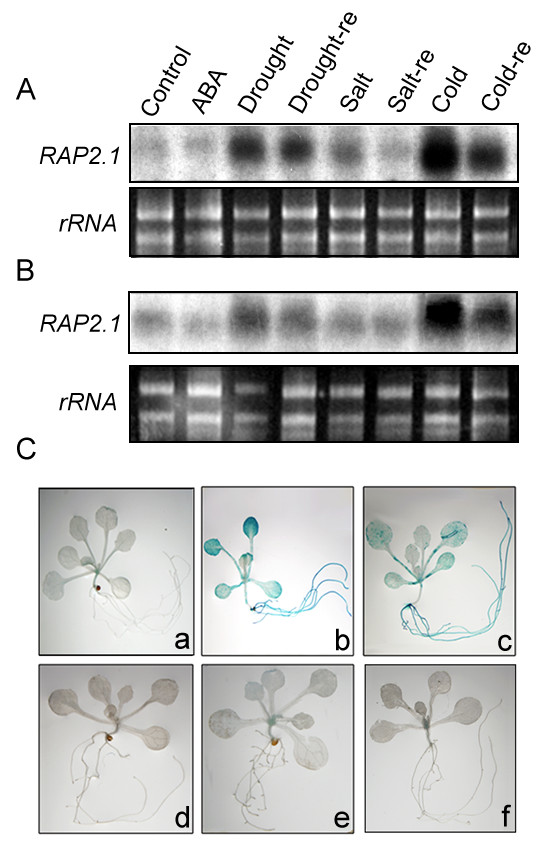
**Regulation of *RAP2.1 *expression by ABA and stress**. A-B, Northern blot analysis with RNA from 2-week-old seedlings of wild type (A) or *aba4-1 *(B). re, 3-h of rehydration after 12-h of stress treatments. (C) Expression of the *RAP2.1p::GUS *reporter gene during stress or ABA treatment. Two-week-old seedlings without any treatment (a) or treated with cold (b), drought (c), high salinity (d), PEG8000 (e) or ABA (f) are shown.

Fowler and Thomashow (2002) showed that transcript levels of *RAP2.1 *exhibited up-regulation at low temperatures by microarray analysis [30]. To investigate *RAP2.1 *expression patterns in response to different abiotic stresses, northern blot analysis was conducted using a gene-specific probe for *RAP2.1*. As shown in Figure 2A, the expression level of *RAP2.1 *was greatly induced by cold and drought stresses, and slightly increased by high salinity stress. In contrast, *RAP2.1 *expression was not influenced by ABA treatment. Similar results were also obtained in the ABA-deficient mutant *aba4-1 *[31], as shown in Figure 2B, indicating that the expression of *RAP2.1 *was governed via an ABA-independent pathway under drought and cold conditions. Interestingly, in all tested Arabidopsis plants, the elevated expression level of *RAP2.1 *resulting from 12-h of drought or cold treatment was reduced by 3-h of rehydration (Figure 2A and 2B).

The promoter sequence of the *RAP2.1 *gene, with a length of 1.5-kb (containing the 5'-UTR), was isolated from the Arabidopsis genome. Histochemical analysis of the *RAP2.1 *promoter-driven β-glucuronidase (*RAP2.1p:GUS*) expression assay is shown in Figure 2C (a-f). The *RAP2.1 *promoter was only responsive to cold (b) and drought (c) stresses, but not to normal conditions (a), high salt stress (d), PEG8000 (e), or ABA (f) treatments. Combining the results from the northern blot and histochemical GUS assays, we conclude that expression of the *RAP2.1 *gene was greatly induced by both cold and drought stresses through an ABA-independent regulatory pathway. This conclusion provides the insight that RAP2.1 may play a critical role in modulating plant responses to drought and cold stresses.

### RAP2.1 negatively regulates drought and cold stresses in Arabidopsis

**Figure 3:**
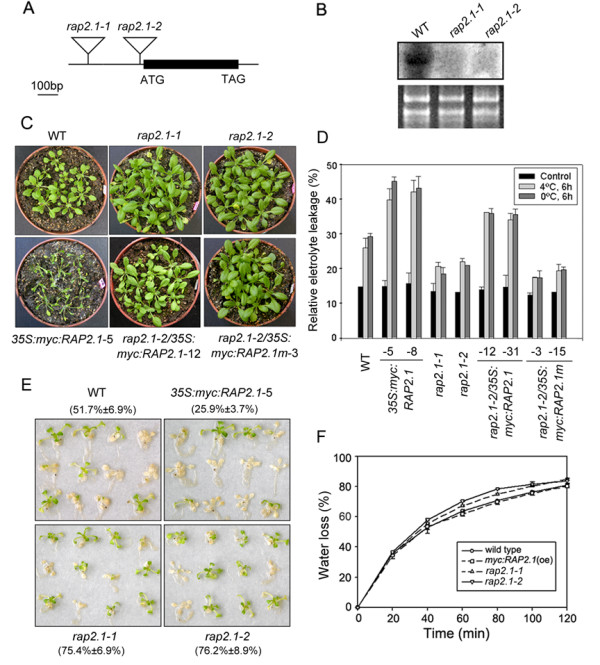
**RAP2.1 negatively regulates plant tolerance to cold and drought stresses**. (A) Schematic structure of *RAP2.1 *mutants, *rap2.1-1 *and *rap2.1-2*. The black rectangle represents the *RAP2.1 *coding region with a single exon. Triangles represent the T-DNA insertions. (B) Northern blot analysis of the *RAP2.1 *gene in wild type (WT), *rap2.1-1 *and *rap2.1-2 *mutants after 12 h of cold treatment. (C) RAP2.1 negatively regulates cold tolerance in Arabidopsis. Representative results of a triplicate independent experiment are shown. (D) Electrolyte leakage from wild type, mutant, and transgenic plants after exposure to low temperature (4°C or 0°C) for 6 h. (E) Photographs are of representative plants with 48 h of rehydration after 8 h of drought treatment. Wild type (WT), *rap2.1-1*, *rap2.1-2*, and *35S:myc:RAP2.1 *(line 5) are shown. Survival rates were determined for at least 40 plants per line. (F) Water loss rate measurement in the wild type, *rap2.1 *mutants and *RAP2.1 *overexpressing plants. Values shown are means of data taken from three independent experiments; error bars indicate SD.

To investigate the *in vivo *role of RAP2.1 in modulating plant responses to drought and cold stresses, "loss of function" and "gain of function" phenotypes of the RAP2.1 protein were identified. For loss of function analysis, we used two Arabidopsis T-DNA insertion mutant alleles of *RAP2.1*, *rap2.1-1 *(SALK_092889) and *rap2.1-2 *(SALK_097874), in which the T-DNAs were inserted into the promoter and 5'-UTR regions of the *RAP2.1 *gene, respectively (Figure 3A). Both *rap2.1-1 *and *rap2.1-2 *were *RAP2.1 *null alleles, showing no detectable *RAP2.1 *transcript in either allele by northern analysis, even after 12-h of cold treatment (Figure 3B). For gain of function analysis, *RAP2.1*-overexpressing transgenic lines (*35S:myc:RAP2.1*) were generated using wild-type plants as background. To perform functional characterization of the EAR-motif of RAP2.1, we also generated a transgenic line expressing a variant of *RAP2.1 *in the *rap2.1-2 *mutant background (*rap2.1-2/35S:myc:RAP2.1m*). This transgenic contained a site-specific mutation that converted the DLNQIP EAR-motif at positions 143-148 to AAAQIA at the same position (as shown in Figure 1A). As a positive control, the transgenic line *rap2.1-2/35S:myc:RAP2.1 *was also generated by expressing the wild type *RAP2.1 *gene in the *rap2.1-2 *mutant background. For each transgenic, at least five independent homozygous lines with high levels of transgene expression (assayed by western blot analysis with anti-myc antibody, data not shown) were identified, and two of these transgenics were randomly selected for subsequent stress tolerance assays.

Firstly, wild-type, mutant and transgenic plants were subjected to cold stress. *RAP2.1 *expression caused increased cold sensitivity, based on growth phenotype (Figure 3C) and relative electrolyte leakage assay (Figure 3D). After 3 weeks of chilling, leaf chlorosis and necrosis were visible in *355S:myc:RAP2.1 *plants (line 5), but could not be detected in wild type plants (Figure 3C). A similar phenotype was also detected in another *355S:myc:RAP2.1 *line (line 8, data not shown). The *RAP2.1 *mutants, *rap2.1-1 *and *rap2.1-2*, displayed significantly better growth than the wild type plants (Figure 3C). The phenotype results were confirmed by a relative electrolyte leakage assay. Electrolyte leakage from *35S:myc:RAP2.1 *plants was approximately 1.5-fold greater than that of wild type plants under either 4°C or 0°C treatment. In contrast, leakage from *rap2.1 *mutants, *rap2.1-1 *and *rap2.1-2*, was only about 70% of that from wild type, even though leakage was similar at the 22°C control temperature (Figure 3D). Expression of the wild-type allele *35S:myc:RAP2.1 *suppressed the cold tolerance of *rap2.1-2 *plants, while the EAR-motif mutated allele *35S:myc:RAP2.1m *could not (Figure 3C and 3D), further confirming that RAP2.1 can function as a negative regulator in plant responses to cold stress and that the EAR-motif of RAP2.1 is directly involved in this process.

Next, the wild-type, mutant and *RAP2.1*-overexpressing plants were further subjected to drought stress. For the 2-week-old seedlings, wild type and *RAP2.1 *mutant plants began wilting 30 min after putting them on dry paper, while the transgenic plants overexpressing *RAP2.1 *could speed up the process, displaying wilt within several minutes. After withholding water for 8 h and rehydration for 48 h, the mutant plants recovered much better (survival ratio of 75.4 ± 6.9% for *rap2.1-1 *and 76.2 ± 8.9% for *rap2.1-2*) compared to the wild type plants (51.7 ± 6.9%), while only about a quarter of the *35S:myc:RAP2.1 *plants survived (25.9 ± 3.7%, for line 5) (Figure 3E). To test whether the altered drought tolerance of the *RAP2.1-*overexpressing plants and *rap2.1 *mutants might be due to leaf transpiration, water-loss rates were measured. As shown in Figure 3F, no significant differences were found between the plants of the three genotypes. A similar phenotype was also detected for another *355S:myc:RAP2.1 *line (line 8) (data not shown). Together, these results suggest that enhanced or reduced drought tolerance of *RAP2.1*-overexpressing or *rap2.1 *mutant plants likely resulted from altered expression of drought-specific responsive genes via an ABA-independent pathway. This would be consistent with the notion that the expression of *RAP2.1 *is up-regulated under drought conditions by an ABA-independent pathway (Figure 2).

### RAP2.1 binds in vivo to the promoters of RD/COR genes and regulates their expression

**Figure 4:**
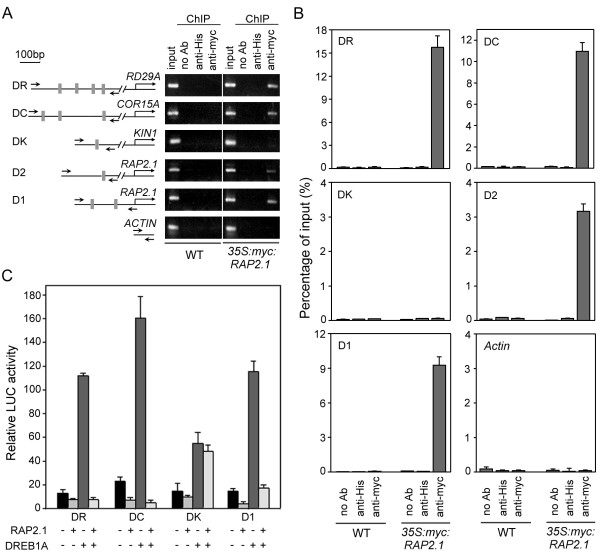
**RAP2.1 binds *in vivo *to the promoters of both *RD/COR *genes and *RAP2.1 *itself, and acts as a transcriptional repressor**. (A) Semi-quantitative PCR from ChIP of samples showed specificity of DNA binding for RAP2.1. Scheme of the gene promoters studied is shown, with gray boxes indicating potential DRE/CRT sites and their positions relative to the putative ATG sites (left panel). The positions of PCR primers used to amplify each fragment were also indicated (small black arrows). Two-week-old seedlings of wild type (WT) and *35S:myc:RAP2.1 *(line 5) were treated with cold for 12 h and then analyzed with or without (no Ab) antibodies specific for the myc-epitope (anti-myc) or the His-tag (anti-His). The *ACTIN *fragment was used as a negative control. (B) ChIP analysis of RAP2.1 binding to promoters in extracts prepared as described in (A) using real-time PCR. (C) Repression of immunoprecipitated fragment-driven reporter gene activity by RAP2.1. Values shown are means of data from three independent experiments; error bars indicate SD.

The transcriptional repression activity of RAP2.1, and the effect of altering *RAP2.1 *expression levels on plant tolerance to cold and drought stresses, suggested that stress responsive genes may be the major targets of RAP2.1 *in vivo*. Previous studies have revealed the presence of DRE/CRTs in the promoters of *RD/COR/KIN (responsive to dehydration/cold-responsive/cold-inducible) *genes, a class of genes up-regulated by cold, water deprivation, salt stress and ABA stimulus [3,9]. We included three genes in our analysis, *RD29A/COR78, COR15A*, and *KIN1*. The distribution of sites and the core sequences of the DRE/CRT elements in the promoters of these three genes, as identified with a plant *cis-elements *database (PLACE, http://www.dna.affrc.go.jp/PLACE/) search, are illustrated in Figure 4A (also see Additional file 1: Table S1).

To determine whether these genes behaved as direct targets of RAP2.1 *in vivo*, we used a chromatin immunoprecipition (ChIP) approach, taking advantage of the cold-treated overexpressing transgenic plants, *35S:myc:RAP2.1 *(line 5), which express a myc-tagged version of RAP2.1. Wild type plants with same treatment were used as a control. Specific immunoprecipition was conducted with an anti-myc antibody and an anti-His antibody was used as a non-specific IgG control. *Actin *was used as a control for the non-DRE fragment. As shown in Figure 4A (left panel), both of the promoter fragments of *RD29A *(DR) and *COR15A *(DC), which contained more than one tandem DRE/CRT, were specifically amplified from the anti-myc immunoprecipitates of *35S:myc:RAP2.1 *extracts (Figure 4A, right panel). However, the *KIN1 *promoter fragment (DK), which contained only one DRE, could not be recovered from the immunoprecipitates with either the anti-myc or the anti-His antibodies. Similar cases were also detected for the *Actin *control fragment. While in the wild type seedlings (WT), there was no myc-tagged protein expressed, and no DNA fragment could be detected from neither anti-myc nor anti-His immunoprecipitates. Additionally, RAP2.1 binding was quantitatively determined using real-time PCR of immunoprecipitates with either anti-HA or anti-myc antibodies. The results fully corroborated the specific binding of RAP2.1 to these promoters *in vivo *(Figure 4B). Both DR and DC fragments included in this analysis showed detectable binding to RAP2.1, while no binding could be detected for the DK and *Actin *fragments.

Next, we carried out a transient expression assay to determine whether RAP2.1 could repress the transcription of the reporter gene driven by the DRE/CRT fragments identified in the ChIP assay. As shown in Figure 4C, the expression results were consistent with the ChIP results. For the LUC reporters driven by the DRE fragments of the *RD/COR *gene promoters, RAP2.1 was able to repress the basal activity of the reporter, as well as expression activity in the presence of an additional transcriptional activator, DREB1A. However, no obvious repression was detected in the reporter driven by the DRE fragment from the *KIN *promoter. These data demonstrate that *RD/COR *genes are likely direct targets of RAP2.1 in *vivo*.

The *RAP2.1 *promoter contains three DRE/CRTs arranged in tandem (Figure 4A and Additional file 1: Table S1). To test whether RAP2.1 could bind to its own promoter *in vivo*, specific primers were used to amplify the DRE fragments of the *RAP2.1 *promoter (D1 and D2) from the ChIP immunoprecipitates. As shown in Figure 4A, both the fragments were detected in the anti-myc immunoprecipitates. The D1 fragment, which contained two DRE/CRTs, was detected at particularly high levels. Furthermore, we determined the binding efficiency of RAP2.1 protein to D1 and D2 fragments by real-time PCR assay. Consistent with the above semi-quantitative PCR results, RAP2.1 was more enriched at the D1 fragment (about 9.25% of input) than D2 (about 3.16% of input), indicating that RAP2.1 binds to D1 fragment with higher efficiency than D2 fragment (Figure 4B). The transient expression assay also showed that the LUC reporter, driven by the D1 fragment, was repressed by RAP2.1 (Figure 4C). This result suggests that RAP2.1 can bind to the DRE elements present in its own promoter and repress its own expression, indicating a negative feedback control in the regulation of expression of *RAP2.1*.

**Figure 5:**
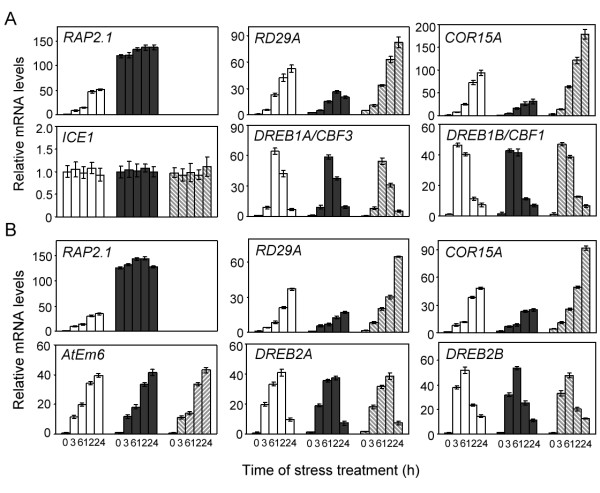
**RAP2.1 is a negative regulator of expression of *RD/COR *genes**. Relative mRNA levels in wild type (white bars), *RAP2.1*-overexpressing (line 5, black bars), or *rap2.1-2 *(hatched bars) plants were determined by real-time PCR. Seedlings were untreated (0 h) or treated with either cold (4°C, A) or drought (B) for the indicated time. Values shown are means of data from three independent experiments; error bars indicate SD.

Since *RD/COR *genes were found to be direct targets of RAP2.1, we used quantitative real-time PCR to determine transcriptional levels of these genes in seedlings of wild-type, *rap2.1-2 *and *35S:myc:RAP2.1 *plants under cold (Figure 5A) or drought (Figure 5B) stresses. In wild-type plants, *RAP2.1 *mRNA accumulation began 6 to 12 h after exposure of the plants to cold (4°C) and reached a maximum expression level at 12 h, after which levels of the transcript were maintained (Figure 5A). Transcript abundance of the *RD29A *and *COR15A *genes slowly and gradually increased over 12 h, reaching a maximum abundance at 24 h after cold treatment. Low temperature-induced transcripts were accumulated to a lesser extent in *RAP2.1*-overexpressing plants than in wild-type. In contrast, transcripts accumulated to greater levels in *rap2.1-2 *seedlings (Figure 5A). The expression of *DREB1/CBF *genes, the upstream regulators of *RD/COR *genes, were induced rapidly (within 15 min) by low temperature in wild-type plants, and transcript accumulation increased with cold treatment [8]. The expression of *DREB1B/CBF1 *preceded that of *DREB1A/CBF3 *(Figure 5A). Furthermore, cold-induced *DREB1/CBF *transcript accumulation was similar in *RAP2.1*-overexpressing and *rap2.1-2 *plants, relative to the control wild type over a 24-h time frame (Figure 5A). A positive regulator of *DREB1/CBF *expression, ICE1 (Inducer of CBF Expression 1) [32], was also detected. *ICE1 *transcript abundance was not affected by cold and was similar in the plants of all three genotypes (Figure 5A). Together, these results indicate that RAP2.1 negatively regulates expression of the *RD/COR *genes and the DREB1/CBF regulons, but does not alter the transcript levels of *DRAB1/CBF*s or *ICE1 *during cold stress.

Similar results were also detected under drought stress. As shown in Figure 5B, drought-induced transcript accumulation of the *RD29 *and *COR15A *genes to a lesser extent in *RAP2.1*-overexpressing plants and to a greater extent in *rap2.1-2 *mutants, relative to the wild type control. The expression of *DREB2A *and *DREB2B *were up-regulated with drought treatment in wild type seedlings, as reported [3,9], and similar expression levels were detected in both *RAP2.1*-overexpressing and *rap2.1-2 *plants (Figure 5B). *AtEm6 *(*Early methionine-labelled 6*), which is regulated by desiccation through a DREB-independent pathway [33], exhibited similar expression patterns in wild type, *RAP2.1 *overexpression and *rap2.1 *mutant plants (Figure 5B). These data indicate that RAP2.1 represses the expression of *RD/COR *genes, but not *DREB2 *genes, under drought stress.

## Discussion

With many DREB-type transcriptional activators having been characterized, the activation mechanisms mediated by DREB proteins involved in plant stress responses are relatively well studied [1,3,8-11,34]. However, sustained activation of plant stress responses during normal growth or in the absence of any stress is metabolically expensive, and runaway responses are apt to induce damage to cellular components [13]. Therefore, plants have evolved repression mechanisms to keep such responses under tight control. A key means of maintaining this control is to use transcriptional repressors to control expression of stress-related genes. We have reported a DREB-type, EAR-motif-containing transcriptional repressor, RAP2.1, which functions as a negative regulator in plant defence responses to cold and drought stresses, maintaining tight control of these responses.

Sequence analysis reveals that RAP2.1 possesses an AP2 DNA-binding domain (Figure 1A). According to amino acid sequence similarity in the AP2 domain, RAP2.1 was classified into the A-5 group of the DREB subfamily [5]. Similar to another characterized member of A-5 group, GhDBP1, RAP2.1 possesses a transcriptional repression domain, the EAR-motif (PDLNxxP) (Figure 1A). In our study, RAP2.1 could indeed repress the basal transcription of LUC reporter genes and the transactivation activity of the transcriptional activator DREB1A (Figure 1D). This finding suggests that RAP2.1 might behave as an active repressor. Multiple possible mechanisms of active repression have been described [35,36], and the mechanism identified from the studies of an EAR-motif-containing repressor, AtERF7 should be informative. AtERF7 binds specifically to the GCC-box and recruits AtSin3 and HDA19, a co-repressor and a histone deacetylase, respectively, to the transcription unit. Deacetylation of histones by HDA19 presumably enhances the binding between the histones and their DNA targets [15]. This kind of repression mechanism through chromatin modification has also been reported for other class II ERF repressors in plants [37,38]. Based on the sequence similarity between the conserved DLNQIP sequence and the EAR-motifs of class II ERFs, RAP2.1 may also recruit such a co-repressor complex to affect repression. Additionally, RAP2.1 could repress transcription in the transient expression assays, where the reporter plasmid is not packaged into chromatin in the same manner as a chromosomal gene. Similar cases also was reported in AtERF7, which could bind to the GCC box and act as a repressor of GCC box-mediated reporter gene transcription in transient expression assay [15]. This indicated that chromatin remodeling may be not the unique repression mechanism for RAP2.1. It may repress the downstream gene expression via other mechanism, such as inhibiting the basal transcription machinery at the specific promoter, or interfering the binding of TBP to the specific TATA boxes [35], or other unknown mechanisms. Therefore, further study is necessary to fully elucidate the complicated repression mechanisms of RAP2.1.

Similar to most EAR-repressors, which are transcriptionally activated by the signals that they negatively regulate [13], *RAP2.1 *transcript was induced by cold and drought stresses (Figure 2). Expression of the *DREB1/DREB2 *genes in response to cold and drought stresses preceded expression of *RAP2.1 *(Figure 5). Considering the DRE-binding and transcriptional repression activities of RAP2.1, we conclude that RAP2.1 acts as a negative "sub-regulon" downstream of the DREB1/DREB2 regulatory pathway [30]. This conclusion was supported by ChIP results, which identified *RD/COR *genes as direct downstream targets of RAP2.1 *in vivo *(Figure 4). Transcript accumulation of *RD/COR *genes under cold and drought stresses could be repressed by *RAP2.1 *expression (Figure 5), thus repressing the plant tolerance to such stresses (Figure 3).

Reasonably, the activation of *RAP.1 *should also be under tight control. We have provided evidence that RAP2.1 can directly repress its own expression, creating a self-inhibitory loop by binding to the DREs present in its own promoter. The existence of feedback loops has been described in various cellular pathways, and they provide for the possibility of buffering, allowing for corrections to the cell system when it is perturbed [39]. In the case of RAP2.1, it is also possible that this negative feedback loop may contribute to oscillatory expression during stress, preventing over-responses to stress treatment.

**Figure 6:**
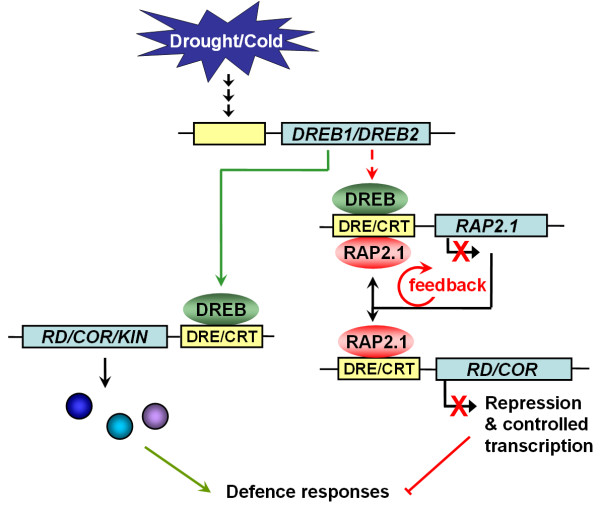
**A possible model for the function of RAP2.1 in modulating the drought and cold stress responses**. Lines with arrows indicate positive regulation and lines with bars indicate negative regulation. The dashed line indicates that there may be an indirect regulatory pathway.

Combining our results and previous findings, we have proposed a hypothetical model for the role of RAP2.1 in modulating plant responses to cold and drought, as presented in Figure 6. Once the cold/drought stress signals arise, *DREB1/DREB2s *are rapidly induced by upstream transcription factors, like ICE1 [32], or others. Next, these transcriptional activators bind efficiently to the DRE/CRT elements in the promoters of downstream genes, such as *RD/COR/KIN *genes, and switch on the DRE-mediated signaling pathway to increase the plants tolerance to the stress. At the same time, DREB1/DREB2s can also up-regulate the expression of *RAP2*.1 in a direct or indirect manner. The induced transcriptional repressor RAP2.1 binds to the DRE/CRT elements upstream of the *RD/COR *genes and represses their expression, thus negatively regulating the plants tolerance to the stress. In addition, the over production of RAP2.1 can be prevented by a negative feedback control mediated by RAP2.1 itself. Therefore, the harmonious operation of the DREB-type activators and the RAP2.1 repressor maintain the activation of the *RD/COR *genes at an appropriate level and the plant stress responses are kept under tight control.

## Conclusion

EAR-motif-containing transcriptional repressors play central roles in the transcriptional regulatory cascades of gene expression in stress response. Runaway stress responses can be prevented through the activity of these repressors. In this study, we reported the *in planta *roles of RAP2.1, an EAR-motif-containing transcriptional repressor, in modulating plant responses to cold and drought stresses in Arabidopsis. *RAP2.1 *transcript accumulated in response to cold and drought stresses. Expression of *RAP2.1 *negatively regulated plant tolerance to cold and drought stress. These stress hypersensitivities were attributable to the repressed expression of the *RD/COR *genes, the *in vivo *direct targets of RAP2.1. Also, we identified a self-inhibitory feedback loop in the expression of *RAP2.1*, which is controlled by RAP2.1 binding to its own promoter and repressing its own transcription. Combining our results, we conclude that RAP2.1 acts as a negative "subregulon" of DREB-type activators and is involved in the precise control of expression of stress-related genes, keeping the stress responses under tight control.

## Methods

### Plant materials and growth conditions

The T-DNA insertion mutants, *rap2.1-1 *(SALK_092889) and *rap2.1-2 *(SALK_097874), were ordered from ABRC (Ohio State University, Columbus, OH). The T-DNA insertion sites were confirmed by PCR and sequencing. Homozygous lines were selected by kanamycin antibiotic resistance and verified by PCR genotyping. For the transgenics, homozygous plants were selected from the T2 generation and confirmed in the T3 generation, based on antibiotic (hygromycin or kanamycin) selection. The ecotype of all plants used in this study was Columbia (Col). Plants were grown on agar plates or in soil in pots in a growth chamber (16 h light and 8 h darkness at 22°C) after stratification for 2 days at 4°C.

### Generation of transgenic plants

For the *35S:myc:RAP2.1 *construct, the *RAP2.1 *coding region was PCR-amplified from Arabidopsis genomic DNA using the primers Fw-RA and Rw-RA (see Additional file 1: Table S2). The PCR fragment was cloned into pCMV-myc (Clontech) for fusion with a c-myc tag at the N-terminal of the RAP2.1 protein. PCR amplification was again used to obtain the myc:RAP2.1 fusion fragment, using the primers Fw-myc (see Additional file 1: Table S2) and Rw-B. The myc:RAP2.1 fragment was cloned into the pMD19-T vector (TaKaRa, JA) for sequence verification, and then sub-cloned as a *Bam*HI/*KpnI *fragment into the modified binary vector pCAMBIA1305 [40]. For the *35S:myc:RAP2.1m *construct, D_143_L_144_N_145_QIP_148 _was replaced with AAAQIA by site-directed mutagenesis using the primers Fw-myc and Rw-RAm (see Additional file 1: Table S2). After verification of the sequence, the PCR product was cloned into the modified pCAMBIA1305. Each of the resulting binary vectors was mobilized into *Agrobacterium tumefacians *GV3101 and transformed into wild-type or *rap2.1-2 *plants by the floral dip method [41]. Hygromycin-resistant transformants were selected and western blots were performed with the anti-myc monoclonal antibody (Clontech).

To generate the *RAP2.1p:GUS *construct, a 1.5-kb fragment of the *RAP2.1 *promoter (including the 5'-UTR) was amplified from genomic DNA using the primers Fw-PB and Rw-PB (see Additional file 1: Table S2). After verification of the sequence, the promoter fragment was digested with *Hind *III and *XbaI *and inserted into the corresponding sites of the pBE2113 vector in place of the *35S *promoter. The resulting *RAP2.1p:GUS *construct was mobilized into *Agrobacterium tumefacians *GV3101 and transformed into wild-type plants. Kanamycin-resistant plants were selected and the homozygous seedlings were used for subsequent histochemical analysis of GUS activities as previously described [41].

### Stress tolerance assays

For the chilling stress assay, wild type, mutant and transgenic plants were grown on germination medium agar plates for 2 weeks then transferred to soil and grown for 1 week at 22°C. The plants were incubated at 4°C for 3 weeks. The plants were photographed and phenotypes were observed. For the survivability tests in dehydration conditions, wild type, mutant and transgenic plants were germinated and grown on MS agar plates for 2 weeks, transferred to Petri dishes, left un-watered for 8 h, and then re-watered. Survival was determined 48 h later. Plants that were green on > 50% of their tissue were considered surviving plants. To minimize the size-dependent effect, plants of similar size were used. All experiments were repeated at least 3 times, with each containing > 40 seedlings per replicate.

### Gel mobility shift assay

Recombinant GST fusion proteins were prepared as described previously [11]. The cDNA fragment encoding the RAP2.1 N-terminal 120 amino acids containing the DNA-binding domain (BD) was inserted into the pGEX6P-1 vector (Pharmacia). The recombinant plasmid was transformed to the *E. coli *strain Rosetta (DE3). Production and purification of the GST fusion proteins were performed as described previously [11]. The 30-bp probe fragments containing the DRE from the *RD29A *promoter with (mDRE) or without (wDRE) a base substitution were synthesized as duplexes, with the sequences shown in Figure 1B. The gel mobility shift assay was performed as described previously [42].

### Transient expression assay

To detect the transactive activity of RAP2.1, a dual reporter system was constructed, as shown in Figure 1C. The reporter plasmids 3×DRE-FLUC and 35S-RLUC (internal control) were constructed as previously reported [42]. For the reporter plasmids with the DRE/CRT-containing promoter fragments used in Figure 4B, the ChIP-detected fragments were PCR amplified and inserted into the 3×DRE-FLUC plasmid in place of the 3×DRE. For the effector plasmids, the construct was similar to that of 35S-RLUC reporter, except that the genes used were *RAP2.1*, EAR-motif-mutated *RAP2.1 *(*RAP2.1m*) or *DREB1A*, instead of RLUC.

The transient expression assay was analyzed in Arabidopsis leaves by particle bombardment as described previously [43]. For each transformation, 10 μg of 3×DRE-FLUC reporter, 0.5 μg of 35S-RLUC reporter, and 10 μg of the effector plasmid were used. After bombardment, the samples were floated on 50 mM phosphate buffer (pH 7.0), incubated at 22°C overnight in the dark, frozen in liquid nitrogen, and LUC activity was quantified with the dual-luciferase reporter assay (Promega).

### RNA analysis

Two-week-old seedlings were harvested from MS agar plates and treated with ABA aqueous solution (100 μM). Or the seedlings were dehydrated on Whatman 3 mm paper at 60% humidity (drought stress). To initiate high salinity stress treatments, the seedlings were placed on Whatman paper soaked with 150 mM NaCl (salt stress). Cold stress was maintained by exposure of plants to a temperature of 4°C. In each case, the plants were subjected to the stress treatments for 12 h, and then re-watered at 22°C for 3 h. The stress-treated seedlings with or without recovery were harvested and frozen in liquid nitrogen. An untreated control was conducted in parallel. Total RNA was isolated using TRIzol reagent (Invitrogen, USA) according to the manufacturer's protocol. RNA gel blot analysis was performed as described previously [29], with the *RAP2.1 *full-length cDNA labeled with [α-^32^P]dCTP as probe.

For the real-time quantitative PCR, 2 μg of total RNA were used as template for first-strand cDNA synthesis using the RNA PCR kit (AMV), version 3.0 (TaKaRa, Japan). Then real-time PCR was carried out using gene-specific primers (see Additional file 1: Table S3) and Power SYBR^® ^Green PCR Master Mix (Applied Biosystems, USA) with a Bio-Rad iCycler iQ system. Each sample was run in triplicate. Relative transcript abundance was calculated using the comparative C_T _method. For a standard control, expression of *Actin *was used. After calculation of ΔC_T _(C_T, gene of interest _- C_T, actin_), ΔΔC_T _[ΔC_T _- ΔC_T, wt (0 h)_] was calculated. The relative expression level was calculated as 2^-ΔΔCT^. A 2^-ΔΔCT ^value for the wild type without cold or drought treatment (0 h) was normalized to 1 [2^-ΔΔCT(ΔCT, wt (0 h) -ΔCT, wt (0 h)) ^= 2^0 ^= 1].

### Chromatin immunoprecipitation (ChIP) assay

The procedure for ChIP of myc:RAP2.1-DNA complex from the wild-type or transgenic Arabidopsis plants was modified from a previous ChIP protocol [44]. Briefly, 2-week-old seedlings were treated with cold (4°C) for 12 h, then were harvested and cross-linked with 1% formaldehyde. The cross-linking reaction was stopped with 0.125 M Glycine. Arabidopsis chromatin was prepared and sonicated to shear DNA to an average size of 500-2000 bp. Crude chromatin lysates were pre-cleared with protein-A agarose beads (Sigma) that were blocked with salmon sperm DNA to prevent non-specific DNA binding. The pre-cleared chromatin samples served as the input controls and were incubated overnight at 4°C either with or without anti-myc or anti-His monoclonal antibodies (Clontech). Immuno-complexes were recovered using protein-A agarose, extensively washed, and eluted from the beads. The samples were treated with proteinase K, the resulted DNA was recovered after phenol/chloroform extraction by ethanol precipitation and dissolved in the dilution buffer (10 mM Tris-HCl, pH 7.5). After immunoprecipitation, recovered chromatin fragments were subjected to semi-quantitative PCR or real-time PCR. Real-time PCR was performed with SYBR-Green-based reagents (Power SYBR^® ^Green PCR Master Mix; Applied Biosystems), using a iCycler iQ real-time PCR Detection system (Bio-Rad). The relative quantities of immunoprecipitated DNA fragments were calculated as the percentage of input chromatin immunoprecipitated using the comparative C_T _method. The primer sequences used are available in Additional file 1: Table S4. Data were derived from three independent amplifications.

### Relative electrolyte leakage assay and water-loss measurement

Relative electrolyte leakage assay of 2-week-old seedlings was performed as described [45], with some modifications. Each seedling of wild-type or transgenic plants was placed into a tube containing 200 μl deionized water. For the 4°C treatment, the tubes were incubated at 4°C for 6 h. For the 0°C treatment, ice chips were added to initiate nucleation and the tubes were incubated in a refrigerated bath at 0°C for 6 h. Deionized water (5 ml) was added to the sample that was then shaken overnight, after which the conductivity of the solution (C1) was determined using a DDS-11A conductivity detector (Kangyi, China). The tube was then incubated at 95°C for 30 min and cooled to room temperature, and the conductivity (C2) of the solution was determined. The values of C1 to C2 were calculated and used to evaluate the relative electrolyte leakage. All experiments were performed with three technical replications, each containing 15 seedlings per line.

For water loss measurement, seedlings of wild-type, mutants and transgenic lines were placed on the weighing dishes and incubated on the laboratory bench. Loss of fresh weight was monitored at the indicated time.

### Accession Numbers

Arabidopsis Genome Initiative locus identifiers for the genes mentioned in this article are as follows: *RAP2.1 *(At1g46768), *RD29A *(At5g52310), *COR15A *(At2g42540), *KIN1 *(At5g15960), *ICE1 *(At3g26744), *DREB1A/CBF3 *(At4g25480), *DREB1B/CBF1 *(At4g25490), *DREB2A *(At5g05410), *DREB2B *(At3g11020), *AtEm6 *(At2g40170).

## Authors' contributions

CJD developed the experimental design, carried out the work, analyzed the data and drafted the manuscript. JYL conceived and coordinated the study, and participated in the experimental design and critically revised the manuscript. Both authors read and approved this final manuscript version.

## Supplementary Material

Additional file 1 **Supplemental materials**. **Figure S1**. Nucleotide and amino acid sequences of RAP2.1. **Table S1**. The distribution of DRE/CRT elements in the promoters of stress genes and the core sequences. **Table S2**. Primers used for construction of vectors with the restriction enzyme sites were underlined. **Table S3**. Primer sequences used to detect genes involved in cold or drought signaling by real-time PCR. **Table S4**. Primer sequences used for ChIP-PCR verification. Click here for file
