# Supplementary material for: The Arabidopsis EAR-motif-containing protein RAP2.1 functions as an active transcriptional repressor to keep stress responses under tight control
Source: BMC Plant Biol. 2010 Mar 16;10:47. doi: 10.1186/1471-2229-10-47 (PMC2848764; doi:10.1186/1471-2229-10-47)
Supplement: Additional file 1 — Supplemental materials. Figure S1. Nucleotide and amino acid sequences of RAP2.1. Table S1. The distribution of DRE/CRT elements in the promoters of stress genes and the core sequences. Table S2. Primers used for construction of vectors with the restriction enzyme sites were underlined. Table S3. Primer sequences used to detect genes involved in cold or drought signaling by real-time PCR. Table S4. Primer sequences used for ChIP-PCR verification. [file 1471-2229-10-47-S1.DOC]

**Additional files:**


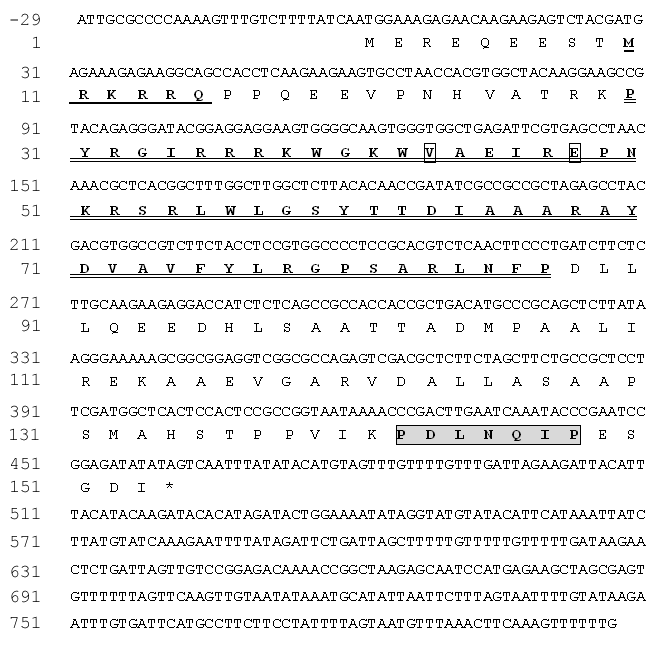


**Figure S1**. **Nucleotide and amino acid sequences of RAP2.1.** Amino acids of the putative nuclear localization signal (NLS) are underlined. The AP2/ERF DNA-binding domain is double underlined, with two conserved amino acids (V and E) shown in open boxes. Also shown is the conserved amino acid residues in EAR-motif, marked using gray boxes.

**Table S1. The distribution of DRE/CRT elements in the promoters of stress genes and the core sequences.**

| **Gene** | **Fragment** | **Location*** | **DRE Site*** | **DRE/CRT Core sequence** |
| --- | --- | --- | --- | --- |
| *RD29A* | DR | -480～-120bp | -208 | AGATCAA**GCCGAC**ACAGACA |
|  |  |  | -240 | GACATGG**ACCGAC**TACTAAT |
|  |  |  | -300 | ATATCAT**ACCGAC**ATCAGTT |
|  |  |  | -352 | ATATACT**ACCGAC**ATGAGTT |
| *COR15A* | DC | -440～-80bp | -140 | TTTCATG**GCCGAC**CTGCTTT |
|  |  |  | -317 | CTTGTTG**GCCGAC**ATACATT |
|  |  |  | -379 | CAACCTT**GTCGGT**TTATTTT |
| *KIN1* | DK | -782～-582bp | -682 | CTACTGA**TCCGAC**ATCAAAA |
| *RAP2.1* | D1 | -258～+27bp | -78 | TATTTAT**GTCGGC**CATAGTT |
|  |  |  | -173 | AATTTCA**GTCGGT**CTCCTAA |
|  | D2 | -703～-348bp | -531 | AAATTTA**ACCGAG**AAGTACT |

List of DRE/CRT elements present in the promoters of stress genes and the core sequences. * The Numbers here indicate the relative positions of DRE/CRTs to the putative ATG codon.

**Table S2. Primers used for construction of vectors.**

| **Primers** | **Sequences** |
| --- | --- |
| Fw-RA | 5’- GAATTCGGGAAAGAGAACAAGAAGAGTC -3’ |
| Rw-RA | 5’- GGTACCCTATATATCTCCGGATTCGG -3’ |
| Fw-myc | 5’- GGATCCATGGCATCAATGCAGAAGCTG -3’ |
| Rw-RAm | 5’- CTATATATCTCCGGATTCGGCTATTTGAGCCGCGGC -3’ |
| Fw-PRA | 5’- AAGCTTGTTCCGAGACTATCCGTG -3’ |
| Rw-PRA | 5’- TCTAGATGATAAAAGACAAACTTTTGGGGC -3’ |

List of primers used in the transgenic construction. The restriction enzyme sites were underlined.

**Table S3. Primer sequences used to detect genes involved in cold or drought signaling by real-time PCR.**

| **Gene** | **Primers** |
| --- | --- |
| *RAP2.1* | Fw-RA2 5’- GGGAAAGAGAACAAGAAGAGTC -3’  Rw-RA2 5’- CTATATATCTCCGGATTCGG -3’ |
| *RD29A* | Fw-RD 5’- TCACTAAACATGGACAAAGCAA -3’  Rw-RD 5’- CAATCTCCGGTACTCCTCCA -3’ |
| *COR15A* | Fw-COR 5’- ATGGCGATGTCTTTCTCA -3’  Rw-COR 5’- CTACTTTGTGGCATCCTTAG -3’ |
| *ICE1* | Fw-ICE 5’- TCCTGGATCTTTGCCTCCA -3’  Rw-ICE 5’- TCAGATCATACCAGCATACCC -3’ |
| *DREB1A* | Fw-1A 5’- AGATCTTGAACTCATTTTCTGCTTTT -3’  Rw-1A 5’- GCGGCCGCTTAATAACTCCATAACG -3’ |
| *DREB1B* | Fw-1B 5’- TCAAGGCGGAGATTATTGTC -3’  Rw-1B 5’- TTAGTAACTCCAAAGCGACAC -3’ |
| *DREB2A* | Fw-1D 5’- ATGGCAGTTTATGATCAGAGTGG -3’  Rw-1D 5’- ATGCTTATCCGCTTTAACACCTC -3’ |
| *DREB2B* | Fw-2A 5’- GAGAAGAGTCTTGTGGAACC -3’  Rw-2A 5’- TCAAATATCCAGAGAACTCAAAC -3’ |
| *AtEm6* | Fw-2B 5’- ATGGCGTCTCAACAAGAGAAG -3’  Rw-2B 5’- TTAGGTCTTGGTCCTGAATTTG -3’ |
| *Actin* | Fw-AC 5’- TTGACTACGAGCAGGAGATGG -3’  Rw-AC 5’- CAAACGAGGGCTGGAACAAG -3’ |

List of primers used in real-time PCR to detect the expression levels of genes involved in cold or drought stress.

**Table S4. Primer sequences used for ChIP-PCR verification.**

| **Gene** | **Primers** |
| --- | --- |
| *RD29A* | Fw-DR 5’- AAAGATCATACCTATTAGAACG -3’  Rw-DR 5’- CTGAGAGAGATAAAGGGACA -3’ |
| *COR15A* | Fw-DC 5’- AATGGTTTGTGGTTTCAAC -3’  Rw-DC 5’- AGGCCACGTGTAATCATA -3’ |
| *KIN1* | Fw-DK 5’- TATCTAGGTATAATTGTGGTTCA -3’  Rw-DK 5’- GAGCCAGACATGTCATATA -3’ |
| *RAP2.1* | Fw-D1 5’- ACTGAGTTTTCGTGATTGTT -3’  Rw-D1 5’- CGTAGACTCTTCTTGTTCTC -3’  Fw-D2 5’- ATAACCCAATGAGGTAAACC -3’  Rw-D2 5’- CATAGATGATGATTGTTTCATAT -3’ |
| *Actin* | Fw-AC 5’- TTGACTACGAGCAGGAGATGG-3’  Rw-AC 5’- CAAACGAGGGCTGGAACAAG-3’ |

List of primers used in PCR and real-time PCR to verify the chromatin fragments obtained from ChIP.
